# Predictors of loss to follow-up after radiotherapy in cancer patients

**DOI:** 10.1007/s00520-026-10486-4

**Published:** 2026-03-03

**Authors:** Cheewathun Pongpradit, Weha Kasemsuk

**Affiliations:** 1 Sawanpracharak Hospital, Nakhon Sawan, Thailand; 2 Department of Public Health Nursing, Faculty of Nursing, Mahidol University, Bangkok, Thailand

**Keywords:** Loss to follow-up appointments, Radiotherapy, Cancer patients, Continuity of care

## Abstract

**Introduction:**

Loss to follow-up after radiotherapy presents a critical challenge in cancer care, undermining treatment effectiveness and efficient use of healthcare resources. Understanding predictors of follow-up non-adherence in the Thai context is essential to improving patient outcomes.

**Research objective:**

This study aimed to determine the rate, underlying causes, and predictive factors associated with loss to follow-up appointments among cancer patients after completing radiotherapy.

**Methods:**

A predictive correlational design was employed with 294 cancer patients who had completed radiotherapy and were scheduled for follow-up appointments. Participants were selected using systematic random sampling. Data were collected through questionnaires, medical record reviews, and telephone interviews for patients who missed appointments. Research instruments included a personal and clinical data form, a radiotherapy service quality assessment based on the SERVQUAL model, and a researcher-developed questionnaire assessing knowledge of follow-up care. Data analysis involved descriptive statistics, chi-square tests, Spearman’s correlation, and multiple logistic regression.

**Results:**

The loss to follow-up rate was 20%. The most common reasons were forgetting appointments (38%), feeling unwell or bedridden (21%), and hospitalization (13%). Multivariate analysis identified two significant predictors: distance from residence to hospital (OR = 1.011, 95% CI 1.003–1.018, *p* = 0.007) and Eastern Cooperative Oncology Group (ECOG) performance status (OR = 1.973, 95% CI 1.355–2.871, *p* < 0.001).

**Conclusion:**

Distance to hospital and poorer physical performance status are key predictors of loss to follow-up. Interventions such as telemedicine, multi-channel reminder systems, and case management for high-risk patients are recommended to strengthen continuity of care and reduce missed appointments.

## Introduction

Cancer remains a pressing global public health concern. In 2022, approximately 19.9 million new cancer cases and nearly 10 million cancer-related deaths were reported worldwide [1]. In Thailand, cancer has been the leading cause of death since 1999, with an estimated 84,073 cancer-related deaths recorded in 2020, averaging 230 deaths per day [2]. The cause of the high cancer mortality rate might be related to accessibility to cancer treatment. These statistics highlight the urgent need for effective and sustainable cancer care strategies. In 2025, Thailand has just launched the “Cancer Anywhere” policy, which covers seven treatments including (1) chemotherapy drugs and hormones, (2) radiotherapy, (3) laboratory fees, (4) treatment for complications arising from cancer treatment, (5) cancer assessment, (6) co-morbidities associated with cancer, and (7) follow-up [3]. Although this policy aims to help cancer patients relieve the burden of treatment expenses, it is limited to patients with universal coverage only.

Radiotherapy is a cornerstone of cancer treatment, utilized by more than half of all cancer patients globally for both curative and palliative purposes [4]. Despite its therapeutic benefits, radiotherapy is associated with short- and long-term side effects that may compromise quality of life. As a result, post-radiotherapy follow-up is essential for ongoing clinical monitoring, timely detection of complications, and effective disease management.

However, loss to follow-up among cancer patients, which is a missing appointment after receiving the full course of radiotherapy, represents a significant barrier to continuity of care. International studies report missed follow-up rates ranging between 10 and 25% [5–7]. In Thailand, monthly statistics from radiotherapy units in 2024 similarly demonstrated loss to follow-up rates between 13.75 and 22.32%. Missed follow-up appointments adversely affect clinical efficiency, resource utilization, and patient outcomes, including survival rates [7, 8]. Disruptions in follow-up care may delay the identification of disease recurrence or treatment-related complications, potentially leading to more complex, costly interventions and a diminished quality of life.

Existing literature categorizes predictors of loss to follow-up into four domains: personal factors (e.g., age, socioeconomic status, education, marital status, and knowledge), geographic factors (e.g., distance to healthcare facilities), clinical factors (e.g., cancer stage, treatment goals, performance status), and healthcare system factors (e.g., service quality, patient satisfaction) [5, 6, 8–11]. While these studies provide valuable insights, they often lack contextual specificity regarding cancer patients in Thailand following radiotherapy. To address this gap, the present study investigates the predictors of loss to follow-up among cancer patients in a Thai tertiary care hospital after completing radiotherapy. Findings from this study are expected to inform the development of targeted strategies aimed at reducing follow-up attrition, thereby enhancing treatment effectiveness, minimizing recurrence risks, and improving healthcare resource allocation.

## Research objectives

1. To determine the rate and underlying causes of loss to follow-up appointments among cancer patients after completing radiotherapy.
2. To identify predictive factors associated with loss to follow-up appointments in this patient population.

## Conceptual framework

Loss to follow-up after radiotherapy is a critical issue that compromises continuity of care, delays detection of complications, and increases the risk of disease progression (Steele et al., 2024). Based on existing literature, the factors associated with missed follow-up appointments can be categorized into four domains: (1) Personal Factors—Age, family income, marital status, educational attainment, and knowledge of post-radiotherapy follow-up, which collectively influence patients’ health-seeking behaviors [5, 6, 8, 10]; (2) Geographic Factors—Distance from residence to hospital, which directly affects travel feasibility, particularly for patients residing in rural or remote areas [6, 11]; (3) Clinical Factors—Diagnosis, cancer stage, treatment goals, time since completion of radiotherapy, and physical performance status, particularly the Eastern Cooperative Oncology Group (ECOG) Performance Status, which reflects functional ability and impacts a patient’s capacity to attend scheduled appointments [6, 10]; (4) Healthcare System Factors—Perceived quality of radiotherapy services, including staff competence, facilities, continuity of care, and patient satisfaction, all of which influence adherence to follow-up recommendations [5, 9].

**Fig. 1:**
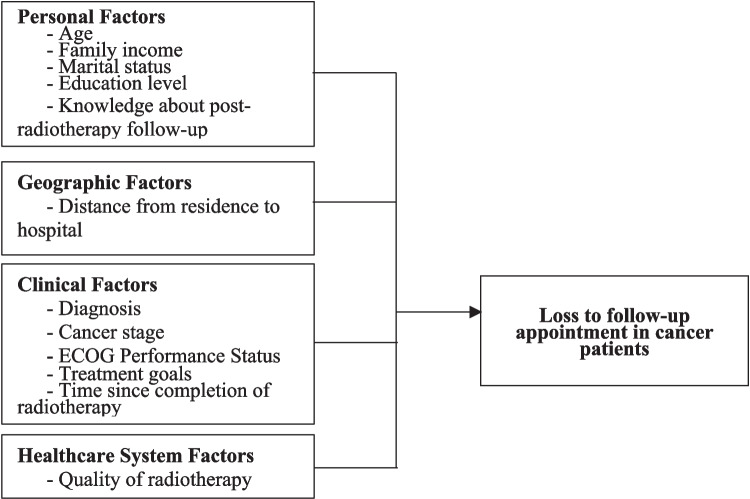
The conceptual framework of this study

These domains collectively informed the conceptual framework guiding the present study (Fig. 1). This framework posits that personal, geographic, clinical, and healthcare system factors interact to predict loss to follow-up among cancer patients post-radiotherapy.

## Research methodology

### Study design

This study employed a predictive correlational research design to determine the rate and causes of loss to follow-up appointments after radiotherapy and to identify predictive factors associated with missed follow-up among cancer patients. The analysis was guided by four domains of potential predictors: personal, geographic, clinical, and healthcare system factors. The study was conducted at the Radiotherapy and Oncology Unit of a tertiary hospital in Thailand.

### Population and sample

The study population consisted of cancer patients who had completed radiotherapy and were scheduled for follow-up appointments at the Radiotherapy and Oncology Unit, Sawanpracharak hospital, a tertiary hospital in the North of Thailand. There are many units involved in taking care of cancer patients including surgical unit, medical unit, ear nose throat unit, gynecology unit, out-patient unit, chemotherapy unit, radiotherapy unit, and interventional radiology unit. From the statistical report of the radiotherapy unit, Sawanpracharak hospital during 2022–2024, there were 9461, 10,175, and 10,767 cancer patients who received treatment from this respectively and, there were new cases of cancer patients for 1169, 1219, and 1252 respectively. The data were collected between January 1 and April 30, 2025. Based on the Cochran’s sample size formula for estimating a population proportion, the required sample size was calculated, yielding 266 participants. To compensate for potential data loss, an additional 10% was added, resulting in a final sample size of 294 patients.

Systematic random sampling was employed to select 10 eligible participants per day from the patient registry. Inclusion criteria were as follows: (1) confirmed any cancer diagnosis which was treated by radiotherapy, (2) completion of radiotherapy courses, (3) age ≥ 18 years, (4) ability to communicate in Thai, and (5) willingness to participate. Exclusion criteria were as follows: (1) critical illness, or (2) severe communication impairments likely to interfere with data collection.

### Research instruments

The questionnaires were developed in a paper-based, self-completion format and consisted of three parts, as follows:

- **Personal, Clinical, and Appointment Data Form** (15 items) which captured demographic variables, clinical information, and appointment-related details, including attendance and reasons for missed appointments.
- **Radiotherapy Service Quality Assessment Questionnaire,** developed based on the SERVQUAL model [12]. The tool contained 16 items across four domains—staff service, environment and facilities, service process, and treatment outcomes/continuity of care—rated on a 5-point Likert scale (5 = Mostly Agreed, 4 = Agreed, 3 = Neutral, 2 = Disagreed, 1 = Mostly Disagreed). Higher scores indicated greater perceived service quality. The instrument demonstrated strong validity (CVI = 1.00) and reliability (Cronbach’s *α* = 0.89).
- **Knowledge and Understanding of Post-Radiotherapy Follow-Up Questionnaire,** developed by the researchers to assess patients’ awareness, understanding, and recognition of the importance, methods, and outcomes of follow-up care after radiotherapy. The questionnaire comprised 16 multiple-choice items, each with four options. Two examples were as follows: “What is the reason for loss to follow-up after completion of radiotherapy course?” and “What is your goal for follow-up after completion of radiotherapy course?” Correct answers were scored as 1 point, and incorrect answers as 0 points, with a possible score range of 0–16. Higher scores indicated greater knowledge and awareness of post-radiotherapy follow-up care. The instrument demonstrated acceptable content validity (CVI = 0.93) and internal consistency reliability (Kuder-Richardson 20 = 0.90).

### Ethical approval

Ethical approval was obtained from the Human Research Ethics Committee of Sawanpracharak Hospital (COA No. 6/2568). All participants provided informed consent. Principles of autonomy, confidentiality, and voluntary participation were strictly upheld throughout the study.

### Data collection procedures

Data collection was conducted in three phases:

1. Preparation Phase: Research instruments were finalized, ethical approval secured, and research assistants trained. To minimize selection bias, a systematic sampling protocol was established using a randomization table. Research assistants were blinded to participants’ clinical outcomes during the recruitment process.
2. Data Collection Phase: Each day, 10 participants were systematically selected from the appointment list using a random starting point and fixed sampling interval to ensure representative sampling across different clinic sessions and days of the week. Eligible participants provided informed consent and completed the questionnaires during their clinic visit. Data collection took approximately 15–20 min. To reduce non-response bias, participants who missed follow-up appointments were contacted via telephone within 48 h. Telephone interviews were conducted after verbal consent was obtained, following the same standardized questionnaire format to maintain consistency. Appointments were rescheduled when possible, and reasons for non-participation were documented.
3. Data Quality Control: Data were checked daily for completeness. Dual entry and cross-verification were performed by two research assistants to ensure accuracy prior to analysis. To minimize reporting bias, all questionnaires were self-administered with research assistants available only to clarify questions without influencing responses. Additionally, a random audit of 10% of entries was conducted by an independent researcher to verify data integrity.

### Data analysis

Data were analyzed using statistical software, SPSS statistics (version 23) under the license held by Mahidol University. Descriptive statistics (frequency, percentage, mean, and standard deviation) were used to summarize participant characteristics. The Kolmogorov–Smirnov test was applied to assess normality of continuous variables. Inferential statistics included Chi-square tests for categorical variables, Spearman’s rank correlation for non-normally distributed continuous variables, and multiple logistic regression (Enter method) to identify predictors of loss to follow-up. Variables with *p* ≤ 0.20 [13] in univariate analysis were included in the multivariate model. Employing a higher *p*-value threshold than the conventional 0.05 helps in not overlooking clinically important variables. These variables may lack statistical significance in a univariate analysis but become relevant predictors when adjusted in a multivariate model. Multicollinearity was assessed using Variance Inflation Factor (VIF). Model fit was evaluated using the Hosmer–Lemeshow test and Nagelkerke *R*^2^. Results were reported as odds ratios (ORs) with 95% confidence intervals (CIs), with statistical significance set at *p* < 0.05.

## Results

### Participant characteristics

A total of 294 cancer patients participated in the study. The majority were female (69%), with a mean age of 59.06 ± 12.17 years (range 25–91), and most were between 51 and 70 years old (59%). Most participants were married (64%), while 23% were widowed, divorced, or separated. Educational attainment was primarily at the primary school level (54%), followed by secondary education (23%).

The mean monthly household income was 24,187.76 ± 35,110.18 baht (range 0–250,000), with one-third (32%) reporting incomes above 20,000 baht. The majority were covered under the Universal Health Coverage Scheme (82%), followed by civil servant benefits (10%) and social security (8%). Nearly three-quarters (76%) reported having a primary caregiver, most commonly a spouse (55%) or a child (31%).

The mean distance from residence to hospital was 54.98 ± 38.48 km (range 2–162). The most common cancer types were breast cancer (33%), uterine/cervical cancer (20%), and head and neck cancer (12%). By stage, 36% were Stage III, 27% Stage II, 24% Stage IV, and 13% Stage I. ECOG Performance Status was 0 in 36%, 1 in 46%, and ≥2 in 18%. Most participants received treatment with curative intent (80%), while 20% were treated palliatively. The mean time since completion of radiotherapy was 17.09 ± 18.06 months (range 1–91). Knowledge regarding follow-up care and perceived service quality were both rated high, with mean scores of 12.44 ± 4.13 and 75.81 ± 5.03, respectively.

### Rate and reasons for missed appointments

Overall, 70% of patients attended their scheduled follow-up appointments, while 20% (*n* = 60) were lost to follow-up. The primary reasons for missed appointments included forgetting the appointment (38%), feeling unwell or bedridden (21%), hospitalization (13%), lack of transportation (10%), refusal of treatment (7%), and conflicting obligations (5%). Less common reasons included feeling recovered (2%), conflicting appointments with other clinics (2%), and scheduling errors (2%). Details are presented in Table 1.

**Table 1:** Reasons of loss to follow-up among participants who lost to their appointment

Reasons of loss to follow-up	*N*	%
Forgot the appointment	23	38
Feeling unwell/unable to attend/bedridden	13	21
Hospitalization	8	13
Nobody available to accompany to hospital	6	10
Refusal of treatment	4	7
Other commitments	3	5
Feeling recovered	1	2
Conflicting appointment with other clinics	1	2
Wrongly scheduled appointment	1	2

#### Bivariate analysis

The analysis of the relationship between the studied factors using the chi-square test revealed that personal factors, including marital status (*χ*^2^ = 1.77, *p* = 0.180) and educational level (*χ*^2^ = 1.06, *p* = 0.300), were not significantly associated with loss to follow-up. Clinical factors, including stage of disease (*χ*^2^ = 0.83, *p* = 0.36) and goal of treatment (*χ*^2^ = 1.82, *p* = 0.180), were also not significantly associated with loss to follow-up, with the exception of ECOG performance status. ECOG performance status was found to be significantly associated with loss to follow-up (*χ*^2^ = 20.64, *p* < 0.001), where participants with an ECOG performance status of 2–4 were more likely to miss appointments than those with a status of 0–1.

The analysis of the relationship between the studied factors using Spearman’s correlation coefficient revealed that the distance from residence to the hospital had a statistically significant negative correlation with loss to follow-up (*ρ* = −0.152, *p* < 0.01). Other factors did not show a statistically significant relationship with loss to follow-up after completion of radiotherapy, including age (*ρ* = 0.027, *p* > 0.05), monthly family income (*ρ* = −0.038, *p* > 0.05), time since completion of radiotherapy (*ρ* = 0.000, *p* > 0.05), quality of radiation therapy services (*ρ* = 0.050, *p* > 0.05), and knowledge and understanding regarding follow-up care (*ρ* = 0.075, *p* > 0.05).

#### Multivariate analysis

Univariate logistic regression is shown in Table 2, identifying distance to hospital and ECOG Performance Status as significant predictors of missed appointments. After adjusting for potential confounders, multiple logistic regression confirmed that distance to hospital was a significant predictor (OR = 1.011, 95% CI 1.003–1.018, *p* = 0.007). Each additional kilometer increased the odds of missed follow-up by 1.1%. ECOG Performance Status was also significant (OR = 1.973, 95% CI 1.355–2.871, *p* < 0.001). Patients with higher ECOG scores were nearly twice as likely to miss appointments compared to those with better functional status. The final regression model was statistically significant (*χ*^2^ = 24.901, df = 6, *p* < 0.001), explaining 12.8% of the variance in loss to follow-up (Nagelkerke *R*^2^ = 0.128). The Hosmer–Lemeshow test indicated good model fit (*χ*^2^ = 13.831, df = 8, *p* = 0.086).

**Table 2:** Multiple logistic regression of predictive factors for loss to follow-up appointments after radiation therapy in cancer patients (*n* = 294)

Variables	*B*	S.E	Wald	*p*-value	OR	95% CI
Family income	0.000	0.000	1.729	0.188	1.000	1.000–1.000
Marital status	−0.146	0.140	1.099	0.295	0.864	0.657–1.136
Distance from residence to hospital	0.010	0.004	7.389	0.007	1.011	1.003–1.018
Cancer stage	−0.042	0.177	0.055	0.815	0.959	0.677–1.358
ECOG	0.679	0.191	12.588	0.000	1.973	1.355–2.871
Treatment goals	−0.120	0.402	0.089	0.766	0.887	0.403–1.951
Constant	−2.217	0.831	7.120	0.008	0.109	-

Cox & Snell *R*^2^ = 0.081, Nagelkerke *R*^2^ = 0.128, *χ*^2^ (6) = 24.901, *p* < 0.001

## Discussion

This study aimed to identify factors associated with loss to follow-up appointments among cancer patients after completing radiotherapy. Findings indicated that ECOG performance status was significantly associated with loss to follow-up. Moreover, two factors emerged as significant predictors of loss to follow-up include distance from residence to hospital and ECOG performance status.

Results found that one fifth of cancer patients missed their scheduled follow-up appointments after completing radiotherapy. This rate is consistent with previous studies, which reported follow-up attrition ranging from 10 to 25% among cancer populations [5–7, 10]. The most frequently reported cause of non-adherence was forgetting the appointment. Forgetfulness is a common determinant of missed visits [14]. The second most cited reason was feeling unwell or bedridden, reflecting the impact of declining physical function inconsistent with the increasing of ECOG performance status. Hospitalization and lack of transportation further underscore the interplay between health status and travelling to hospital issue to receive care.

The distance between a patient’s home and the hospital was strongly associated with loss to follow-up among individuals with cancer. Those living farther from treatment centers were more likely to miss follow-up appointments [6, 11]. Although Thailand’s “Cancer Anywhere” policy enables patients to access cancer-related care at any public hospital without additional charges [15], geographic barriers remain. Travel time, indirect expenses, and disruptions to daily routines continue to hinder access, as the policy does not cover transportation costs. These challenges underscore the importance of decentralizing follow-up services or expanding oncology care within community settings.

Clinical factors also played an important role. Patients with poorer ECOG Performance Status were nearly twice as likely to miss follow-up compared to those with better functional capacity. Patients experiencing greater treatment-related side effects or functional decline were less able to adhere to follow-up schedules [9, 10]. This suggests that mobility limitations and deteriorating health remain critical barriers, even when healthcare access is financially supported.

This study found that personal factors, including age, family income, marital status, education level, and knowledge and understanding regarding follow-up symptoms, were statistically insignificantly associated with missed follow-up appointments. This contradicts previous studies which found that younger cancer patients, patients with low income [8], unmarried patients [6] and cervical cancer patients with a low education level [10] were more likely to miss appointments.

These inconsistencies can be attributed to distinctive features of Thailand’s healthcare system and social context. First, only a small proportion of participants bore the cost of medical expenses themselves, as nearly all received cancer treatment free of charge. Most were covered under the Universal Coverage healthcare scheme, which provides comprehensive cancer care—from diagnosis through follow-up—thereby minimizing financial barriers to radiation therapy. In addition, the “Cancer Anywhere” initiative enhances equity by enabling cross-regional access to services without extra costs [15]. Second, Thailand’s strong extended family structure plays a crucial role in mitigating the impact of marital status on healthcare access [16]. This study revealed that primary caregivers were often children, siblings, or close relatives, reflecting the deeply rooted kinship-based caregiving culture. This differs markedly from Western and African contexts, where elderly or unmarried patients often encounter greater challenges in travel and healthcare access [10]. Third, the hospital’s integrated care system—with coordinated medical staff, structured appointment scheduling, and consistent patient education—contributed to high levels of knowledge and awareness regarding post-radiation follow-up symptoms. Moreover, the hospital’s practice of conducting follow-ups via telephone further reduced the influence of educational background on patients’ understanding of the importance of follow-up care. While global predictors of follow-up adherence are well-documented, the Thai healthcare landscape—characterized by the “Cancer Anywhere” policy and a concentrated distribution of radiotherapy facilities—presents unique logistical challenges. Understanding these local factors is crucial for tailoring interventions that fit the Universal Health Coverage (UHC) framework in Southeast Asia.

For other clinical factors, the stage of disease was statistically insignificantly associated with lost to follow-up appointments, which is consistent with the study by Akufuna et al. [9] that found the stage of disease in cervical cancer patients did not affect the continuity of receiving radiation therapy services. However, this differs from the studies by Habinshuti et al. [10] and Morris et al. [11], which found that patients with advanced-stage cancer were more likely to miss follow-up appointments than those in the early stage. This inconsistency may arise from the specific characteristic of the sample, which mostly consisted of breast cancer patients, who have different follow-up behaviors than other types of cancer patients, with a high appointment attendance rate [17]. This is because breast cancer patients are more aware of the importance of monitoring for recurrence, as breast cancer has a risk of recurrence that may last for many years [18]. Additionally, the majority of the sample had an ECOG performance status of 0–1, reflecting that patients have appropriate physical capacity and can perform daily activities normally. Therefore, even if the patients are in stage 3–4 of the disease, they still have the ability to continuously travel for follow-up.

Time since completion of radiation therapy was statistically insignificantly associated with missed follow-up appointments, which contradicts the studies by Hoyle et al. [6] and Steele et al. [7] that found missed appointments tended to increase as the time since the end of treatment was longer. This inconsistency may result from the characteristic of the sample having an average time since completion of radiation therapy of 17.09 months, which is still within the period where doctors and patients prioritize monitoring for late-stage side effects from radiation and disease recurrence. Moreover, the efficient continuous care system, the Thai cultural factor emphasizing obedience to doctor’s advice, and strong family support may help maintain a stable follow-up attendance rate, even as time passes since the end of treatment.

This study found that service quality was statistically insignificantly associated with missed follow-up appointments, which contradicts the study by Akufuna et al. [9]. This inconsistency may stem from the characteristic of the sample, who consistently gave high scores for radiation therapy service quality, which may reflect the quality management system of the healthcare facility as a tertiary hospital with consistent service standards. Furthermore, missed follow-up appointments may primarily be caused by factors external to the service system, such as personal factors (forgetting appointments, no one to transport them) and health factors (unwell/unable to attend), which accounted for more than 70% of the reasons for missed appointments in this study.

## Recommendations for research implementation

The findings of this study suggest the need for practical interventions that address both structural and clinical barriers to follow-up care after radiotherapy. First, the implementation of telemedicine systems should be prioritized to provide remote consultation and monitoring for patients residing in distant or rural areas. Complementary reminder systems, such as SMS or LINE notifications, can be integrated into hospital workflows to minimize missed appointments due to forgetfulness. Second, patients with poor ECOG Performance Status should be targeted for intensive case management. This may include structured discharge planning, caregiver engagement, and home visit programs to ensure continuity of care despite physical limitations. Third, decentralizing follow-up services to community-level or provincial hospitals could reduce travel burdens and enhance accessibility, particularly for patients in underserved regions. Strengthening interdepartmental coordination for timely rescheduling of missed appointments may further improve adherence. Together, these strategies can enhance continuity of care, reduce recurrence risks, and optimize healthcare resource utilization.

## Recommendations for future research

Future research should evaluate the effectiveness of interventions designed to mitigate follow-up attrition, such as telemedicine platforms, multi-modal reminder systems, and decentralized oncology care services. Rigorous trials assessing their clinical outcomes and cost-effectiveness would provide critical evidence for policy integration. Additionally, longitudinal studies are warranted to explore how predictors of follow-up adherence evolve over time, particularly across different cancer subtypes and stages. Comparative studies between urban and rural populations could further clarify the influence of geographic barriers under Thailand’s universal health coverage system. Finally, qualitative investigations exploring patient and caregiver perspectives may provide deeper insights into psychosocial and contextual factors influencing follow-up behaviors. Such research would complement quantitative findings and support the development of holistic, patient-centered care models that improve long-term cancer outcomes.

## Conclusion

This cross-sectional study examining loss to follow-up after radiotherapy among cancer patients in a tertiary hospital in Northern Thailand highlights that structural barriers and clinical limitations—rather than socioeconomic status or service quality—are the main drivers of follow-up discontinuity in the Thai setting. Although universal health coverage and strong family support help reduce financial and social obstacles, continuity of care is still hindered by geographic distance and declining physical function. To address these challenges, interventions such as telemedicine, appointment reminder systems, decentralized follow-up services, and targeted case management for high-risk patients are crucial to enhance adherence, improve treatment outcomes, and promote equitable access to cancer care.

## Limitations

This single-center study at a tertiary hospital with predominantly breast cancer patients (33%) and universal health coverage beneficiaries (82%) may limit generalizability to other settings and cancer populations. The cross-sectional design with variable follow-up periods (1–91 months post-radiotherapy) and exclusion of critically ill patients potentially introduced selection bias and underestimated true loss to follow-up rates. The modest explanatory power of the predictive model (Nagelkerke *R*^2^ = 0.128) suggests substantial unmeasured factors—including psychological variables, transportation availability, and financial constraints beyond direct medical costs—that influence appointment attendance. Additionally, reliance on self-reported data and telephone interviews may have introduced recall and social desirability biases, while the newly developed instruments, despite acceptable reliability, require further validation across diverse populations.

## Data Availability

No datasets were generated or analysed during the current study.
